# The Development of Complex Digital Health Solutions: Formative Evaluation Combining Different Methodologies

**DOI:** 10.2196/resprot.9521

**Published:** 2018-07-16

**Authors:** Anne Lee, Marianne Sandvei, Hans Christian Asmussen, Marie Skougaard, Joanne Macdonald, Jakub Zavada, Henning Bliddal, Peter C Taylor, Henrik Gudbergsen

**Affiliations:** ^1^ Centre for Innovative Medical Technology Odense University Hospital Odense Denmark; ^2^ Research Unit of User Perspectives Department of Public Health University of Southern Denmark Odense Denmark; ^3^ The Parker Institute Copenhagen University Hospital, Bispebjerg and Frederiksberg Frederiksberg Denmark; ^4^ Botnar Research Centre Nuffield Department of Orthopaedics, Rheumatology and Musculoskeletal Sciences. University of Oxford Oxford United Kingdom; ^5^ Institute of Rheumatology Prague Czech Republic; ^6^ Charles University, First Faculty of Medicine Department of Rheumatology Prague Czech Republic

**Keywords:** eHealth, telemedicine, stakeholder participation, formative evaluation, participatory design, intervention theories

## Abstract

**Background:**

The development of digital health solutions for current health care settings requires an understanding of the complexities of the health care system, organizational setting, and stakeholder groups and of the underlying interplay between stakeholders and the technology. The digital health solution was founded on the basis of an information and communication technology platform and point-of-care devices enabling home-based monitoring of disease progression and treatment outcome for patients with rheumatoid arthritis (RA).

**Objective:**

The aim of this paper is to describe and discuss the applicability of an iterative evaluation process in guiding the development of a digital health solution as a technical and organizational entity in three different health care systems.

**Methods:**

The formative evaluation comprised the methodologies of contextual understanding, participatory design, and feasibility studies and included patients, healthcare professionals, and hardware and software developers. In total, the evaluation involved 45 patients and 25 health care professionals at 3 clinical sites in Europe.

**Results:**

The formative evaluation served as ongoing and relevant input to the development process of the digital health solution. Through initial field studies key stakeholder groups were identified and knowledge obtained about the different health care systems, the professional competencies involved in routine RA treatment, the clinics’ working procedures, and the use of communication technologies. A theory-based stakeholder evaluation achieved a multifaceted picture of the ideas and assumptions held by stakeholder groups at the three clinical sites, which also represented the diversity of three different language zones and cultures. Experiences and suggestions from the patients and health care professionals were sought through participatory design processes and real-life testing and actively used for adjusting the visual, conceptual, and practical design of the solution. The learnings captured through these activities aided in forming the solution and in developing a common understanding of the overall vision and aim of this solution. During this process, the 3 participating sites learned from each other’s feed-back with the ensuing multicultural inspiration. Moreover, these efforts also enabled the consortium to identify a ‘tipping point’ during a pilot study, revealing serious challenges and a need for further development of the solution. We achieved valuable learning during the evaluation activities, and the remaining challenges have been clarified more extensively than a single-site development would have discovered. The further obstacles have been defined as has the need to resolve these before designing and conducting a real-life clinical test to assess the outcome from a digital health solution for RA treatment.

**Conclusions:**

A formative evaluation process with ongoing involvement of stakeholder groups from 3 different cultures and countries have helped to inform and influence the development of a novel digital health solution, and provided constructive input and feedback enabling the consortium to control the development process.

## Introduction

Digital health solutions involve the use of telecommunication and virtual technology to deliver health care outside the traditional health care facilities [1]. The importance of digital health solutions as a vehicle for delivering timely care over distance is on the rise due to increased health needs that have overwhelmed health care sectors across the globe [2]. The widespread use of information and communication technologies (ICTs) in daily life and the growing focus on patient-centered care and self-management strategies have promoted an acceptance of moving health care delivery from the established health care facilities to solutions that come directly into peoples’ homes [3-5].

Rheumatoid arthritis (RA) is a chronic, inflammatory disease that leads to joint damage and loss of physical function, and it affects about 3.5 million individuals across Europe [6]. Current clinical care includes assessment of the patient’s joints, analysis of blood samples, and reviewing patient-reported outcomes (PROs). The wide range of treatment options and increasingly specific treatment goals has made the management of RA difficult, and traditional monitoring methods typically involve visits to the hospital that are both time-consuming and resource-intensive.

The Horizon 2020 project eHealth in Rheumatology (ELECTOR) was launched in 2014 with the aim of developing and implementing a digital health solution founded on the basis of an ICT platform for home-based monitoring of disease progression and treatment outcome for patients with RA. The goal of this project was to develop a digital health solution as an alternative to some of the standard visits to the hospital outpatient clinic. The project was a public-private collaboration that included 3 outpatient clinics in the United Kingdom, the Czech Republic, and Denmark and an international team of hardware and software developers, clinicians, designers, and researchers from the Netherlands, the UK, the Czech Republic, and Denmark [7].

**Figure 1:**
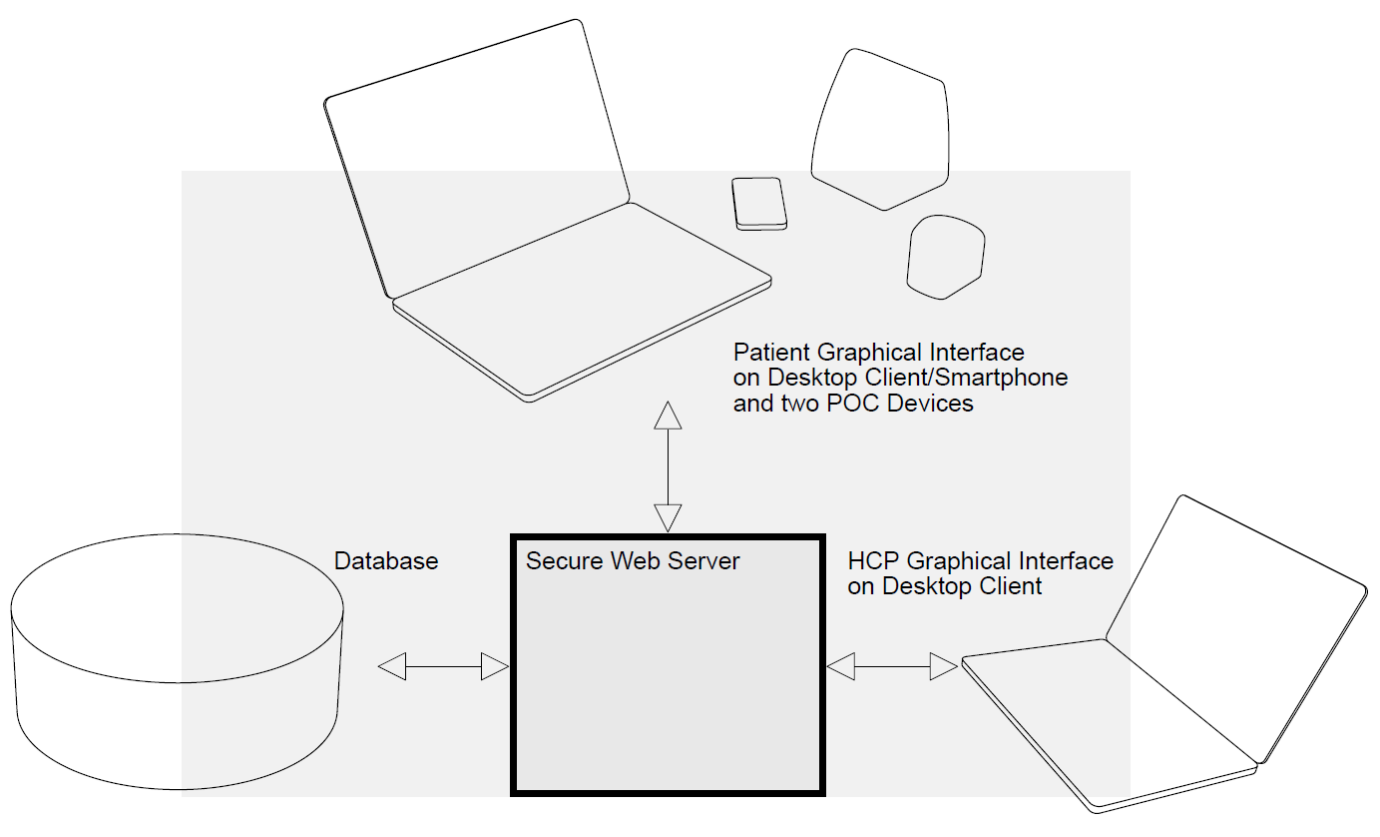
The ICT platform enabling open-source data storage and exchange through secure internet access to a web-based graphical user interface for patients and healthcare professionals (HCPs). PoC: point-of-care.

In the ELECTOR project, the concept for home-based monitoring of patients with RA encompasses an ICT platform and point-of-care (PoC) devices as well as new methods of organizing outpatient consultations, as illustrated in Figure 1. The development of the solution was guided by a formative evaluation using a range of methodologies and involving key stakeholders across 3 health care settings in Europe. The aim of this paper was to describe and discuss our experiences from this 2-year evaluation process.

## Methods

### Study Design

The ICT platform enables open-source data storage and exchange through secure internet access to a web-based graphical user interface for patients and health care professionals. The platform is available on computer, tablet, and smartphone as a “bring your own device” initiative. The ICT platform enables video communication, reporting of PRO data, and connection to PoC devices that can analyze blood samples at the patient’s home.

Several different blood parameters such as high sensitivity C-reactive protein (hsCRP), alanine aminotransferase (ALT), white blood cell (WBC), and hemoglobin (Hgb) are tested to monitor RA and its treatment, and as none of the available PoC devices can perform all these tests, 2 new PoC devices were used. The PoC device for analyzing WBC and Hgb is not yet approved for patient use and thus managed by health care professionals attending the patient at home. The PoC device for analyzing hsCRP and ALT is under development.

With the ICT platform was in place, patients made an assessment of their joints and used the PoC device to analyze blood samples. They also completed several PRO questionnaires and send the data to the hospital. At the outpatient clinic, the health care professionals assessed the data and responded to the patients accordingly via synchrony (video) or asynchrony (mail). Background information and instruction material were required for both patients and health care professionals.

Development of the digital health solution was guided by a formative evaluation engaging a consortium that was established to ensure the development of a solution that would be applicable in all the 3 different health care systems. The consortium included the project coordinator, clinical researchers from the 3 clinical sites, software and hardware developers, and an evaluation team. The outcome of each evaluation activity was shared within the consortium through written reports, oral presentations, and discussions at regular meetings to optimize mutual understanding and insight into the ICT platform and its components.

The digital health solution was developed in 3 languages, and variations in culture as well as treatment traditions were to be reflected in the adaptability of a coming solution. Patients, health care professionals, and the local outpatient clinic managers were identified as end users of the technology and thus the key stakeholders to be included in the evaluation. Patients were recruited through affiliated research departments, and we aimed to include a diverse group of patients in terms of sex, age, duration of RA, distance to the outpatient clinic, and socioeconomic status. Health care professionals were recruited from the 3 participating clinics and included both physicians and nurses. A total of 45 patients, 25 health care professionals, and local managers from each of the 3 clinics participated in the evaluation.

The study activities were approved as required by the local health authorities in the participating countries. Study participants received written and oral information about the study and provided written consents.

### Evaluation Methodologies and Activities

**Figure 2:**
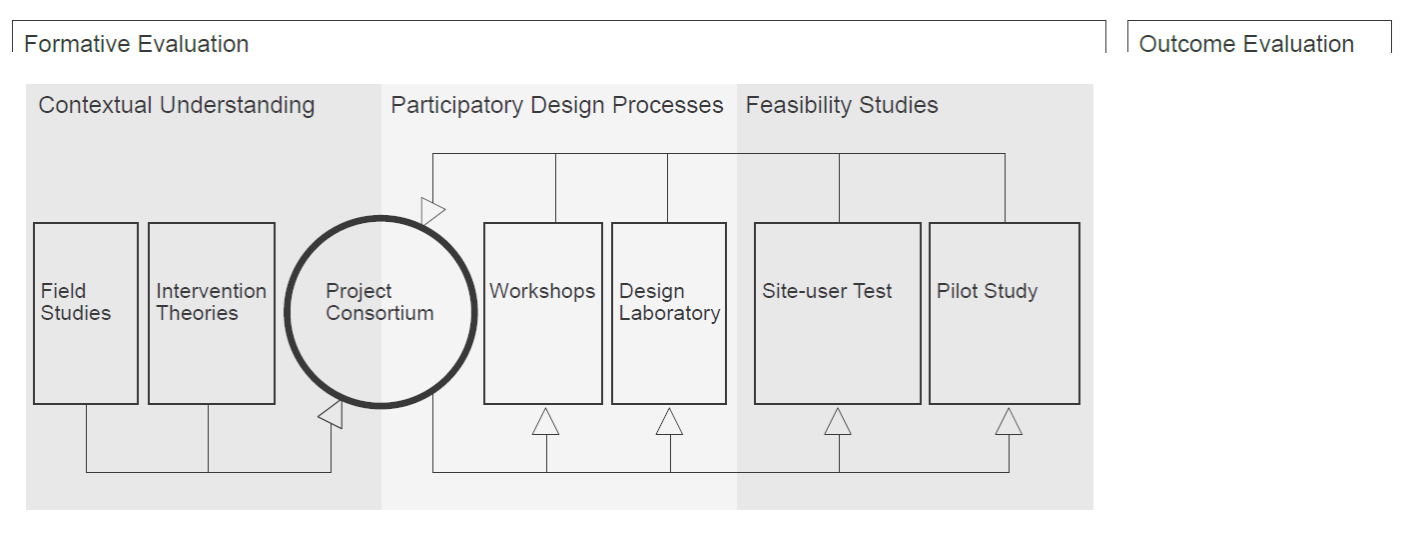
Methodological approaches and activities used to evaluate the development of the eHealth concept.

The formative evaluation included the following 3 primary methodologies: (1) contextual understanding, (2) participatory design processes, and (3) feasibility studies. Each of these contained several activities that were designed along the way as the need for new insights emerged and because the outcome of one activity influenced the subsequent activity. An overview of the primary methodologies and activities is presented in Figure 2.

### Contextual Understanding

Before initiating the development process, we conducted field studies and a theory-based stakeholder evaluation with the overall aim of gaining contextual knowledge about the routine outpatient management of RA and the ideas and assumptions among stakeholder groups.

#### Field Studies

In March and April 2015, we conducted 2 days of field studies at each of the 3 clinical sites to gather information about the clinical context, identify the stakeholder groups, and engage the clinical sites in the development of the digital health solution.

We observed daily clinical practices and held informal interviews [8] with health care professionals and patients. Field notes were made during the clinic visits and were used to describe the clinical sites, key stakeholder groups, and clinical routines in the outpatient treatment of RA.

#### Intervention Theories

From April to October 2015, we conducted a theory-based stakeholder evaluation to achieve a multifaceted picture of the ideas and assumptions held by the stakeholder groups. This was a combination of an intervention theory approach and a stakeholder approach aimed at improving evaluation practice and intervention quality when various stakeholders are assumed to be decisive for the implementation and functioning of an intervention. Intervention theories are defined as presuppositions of how an intervention may impact a given situation by changing or preserving it in ways that are preferable or not preferable to a situation without the intervention or with another intervention [9].

Initially, we developed the common intervention theory or the so-called “principle of reason” for the concept. This was based on project descriptions, presentations of the digital health solution, written material describing the current clinical care, and discussions within the project consortium. Next, we developed intervention theories for each key stakeholder group. From August to October 2015, we held group and individual interviews with a total of 16 patients and 22 health care professionals at the 3 clinics (Table 1).

A semistructured interview guide was used, and a prototype of a PoC device served as a prompt during interviews. The interviews were audiotaped and transcribed, and the transcripts were categorized into the following predetermined themes: (1) situation theory (interpretations of present challenges), (2) normative theory (perceptions of the preferred or ideal situation), and (3) causal theory (assumptions of how the intervention works). A description of each theme was then formulated for each stakeholder group and presented in a matrix comparing the principle of reason theory with the intervention theories of the patients and health care professionals at each clinical site. This allowed the similarities, differences, and possible conflicts between stakeholder groups to become apparent (Multimedia Appendix 1).

### Participatory Design Processes

The aim of the participatory design approach was to develop the technical systems and the related organization in close cooperation with end users early in the design and development process and in a contextualized and realistic setting [10-12]. The participatory design process included workshops at each of the 3 clinical sites as well as a design laboratory facility established at the Copenhagen site.

#### Workshops

One workshop was held at each of the clinical sites between August and October 2015. The aims of the workshops were to gain knowledge about the overall usability of the graphical user interface and PoC devices and to gain knowledge about patients’ and health care professionals’ perceptions of the digital health solution. In total, 10 patients and 18 health care professionals participated in the workshops (Table 1). Each workshop included “hands-on” sessions and “think aloud” methods [13] in small groups of patients and health care professionals, as well as discussions of the advantages and disadvantages of the solution in groups of patients and health care professionals and in plenary.

Hands-on sessions included the following steps: (1) patients conducting a monitoring session while the health care professionals observed, that is, logging on to the system, completing the PRO questionnaires, and filling in the (predesigned) blood test results, 2) health care professionals logging on to the system and receiving data while the patients observed, 3) patients and health care professionals conducting a virtual consultation. During the sessions, the patients and health care professionals were encouraged to comment on what they experienced (think aloud) and share their thoughts afterward.

Following the hands-on sessions, the groups of patients and health care professionals discussed perceived strengths and possibilities and weakness and challenges of the solution. These discussions were audiotaped and transcribed. Transcriptions and notes taken during the sessions were compiled as text, photos, and drawings.

#### Design Laboratory Facility

The design laboratory was established in 2015 as a “home-like setup” in an apartment at the Copenhagen clinic. Its aim was to help developers to answer specific questions such as “*Does this device or process work and how can it be refined?* ”

In a series of short sessions, prototypes of PoC devices, lancets for drawing blood, and cartridges for blood sampling were tested through a dialogue between the patient and designer while iteratively sketching and evaluating prototypes and different scenarios for blood testing (Table 1). These sessions were observed by developers and a researcher taking notes and photos and interviewing patients.

**Table 1:** Evaluation activities performed between 2015 and 2017.

Activity	Aim	Place and participants	Data collection	Outcomes
Field studies, Primo 2015	Gaining knowledge of contexts and daily practice, creating collaboration, and identifying key stakeholders.	Outpatient clinic at the 3 clinical sites. Actors involved in the work at the clinics.	Observations and informal interviews with health care professionals and clinic management.	Descriptions of daily clinical practices as input for identifying stakeholders and further evaluation.
Intervention theories, Medio 2015	Developing intervention theories for key stakeholder groups.	At the 3 clinical sites. 16 patients (aged 25-75 years, 11 females) and 22 health care professionals (16 females), including 3 clinic managers.	Semistructured interviews (individual and group).	Descriptions and comparisons of intervention theories across stakeholder groups. Report served as input to the ongoing development of the digital health solution.
Workshops, Medio 2015	Insights into patient and health care professionals’ perceptions of the digital health solution and overall usability of the graphical user interface.	At the 3 clinical sites. 10 patients (aged 26-69 years, 6 females) and 18 health care professionals (13 females).	“Hands-on” sessions and “think aloud” exercises in small groups of patients and health care professionals. Group and plenary discussions of pros and cons of the digital health solution.	Presence of developers enabled immediate adaptations. Reports served as input to the technical and the organizational development as well as the clinical value creation.
Design laboratory, Ultimo 2015	Testing how patients handled different lancets and cartridges for blood sampling.	At the design laboratory. 2 patients (1 female).	Patients drew a drop of blood and filled a cartridge. Sessions included observation, dialogue, drawing, and generation of ideas.	Recommendations and optimizations in a fast turnover. Provided valuable input to the design and development of point-of-care devices and related test cartridges.
Site user tests, Primo 2016	Testing the “lifecycle management” of the technology.	At the 3 clinical sites and the patient’s home. 15 patients (aged 25-83 years, 9 females) and 3 health care professionals (3 females).	Home monitoring for 1 week. Log data from the information and communication technology (ICT) platform, notes from health care professionals, diary, and photos from patients.	Descriptive analysis reported for each site, resulting in revisions of the technical and organizational setup of the digital health solution and the related briefing and communication material.
Design laboratory, Medio 2016	Test of different scenarios for blood testing.	At the design laboratory. 4 patients (3 females).	Following a short introduction, patients performed a blood test. Sessions included observation, dialogue, drawing and generation of ideas, and interviews.	Descriptive analysis reported. Served as input to the organizational development.
Pilot study, Ultimo 2016	To test real-life functionality of the digital health solution.	At the clinical site in Copenhagen and the patient’s home. 5 patients (aged 55-83 years, 4 females) and 1 health care professional (female).	Home monitoring for 3 weeks. Log data from the ICT platform, notes from health care professionals, diaries, and photos from patients. Subsequently, interviews with the 5 participating patients.	Interim analysis reported. Indicated a “tipping point” and a need for further development.

### Feasibility Studies

The feasibility studies included a site user test at each clinical site and a pilot study at the Copenhagen site. These were completed during 2016. The feasibility studies were used to test if an intervention could be performed in a particular setting and to investigate how contextual factors influenced the implementation of the technology. The aim was to provide feedback to the developers to create learning and a basis for adapting the technology to its actual users and the implementation context [14].

#### Site User Tests

The aim of the 1-week site user test was to assess the “life cycle management” of the concept, that is, the practical aspects of handing out, taking home, and installing the ICT platform components. At the clinic, the patients received oral instructions on how to perform joint assessment, how to connect to the ICT platform, and how to communicate with the clinic. The prototypes of PoC devices, written information, a Polaroid camera, and a notebook were then handed out to the patients. At home, patients connected to the ICT platform from their own computer using a secure log-in. Every second day, they completed a joint assessment and the PRO questionnaires and entered the results of the blood tests and also video-communicated with the health care professionals at the hospital. A total of 15 patients and 3 health care professionals participated in the site user tests (Table 1).

Data collected from the site user tests included log data from the ICT platform and health care professionals’ notes during the test. Patients’ experiences were collected via a cultural probe [15] consisting of a notebook, a Polaroid camera, and a leaflet explaining the intended use for capturing patients’ experiences with the digital health solution. Based on these data, descriptive analyses were made focusing on the patients’ and health care professionals’ attitudes toward the home monitoring concept and experiences with the solution, including technical and organizational issues as well as issues related to task performance.

#### Pilot Study

The pilot study was planned as a 3-week pilot study with 30 patients and was conducted at the clinical site in Copenhagen from December 2016 to January 2017. The aim of the pilot study was to test the “real-life organizational setup” of the concept, and it served as a base for a subsequent outcome evaluation (Table 1).

After getting instructions at the hospital, patients brought home the leaflet outlining the intended use along with the 2 PoC devices, which were then connected to the ICT platform from their own PC using a secure log-in. Every second day, they performed home monitoring, including joint assessment, PRO questionnaires, blood testing (predefined results were used), and virtual contact with the hospital.

Data included compliance with scheduled activities, use of a hospital hotline for technical and health-related inquiries, and experiences of patients and health care professionals retrieved through notes, diaries, photos, and patient interviews.

## Results

### Contextual Understanding

The initial field studies enabled us to identify key stakeholder groups and provided us with knowledge about the different health care systems, professional competencies involved in routine RA management, clinics’ working procedures, and use of communication technologies. For example, while RA management in Denmark is hospital-based and patients can choose to have blood tests at hospital or the general practitioner’s clinic, RA management in England is collaboration between hospitals providing RA consultations and general practitioners responsible for undertaking blood tests and prescribing medications. In CZ, RA treatment is primarily located in a single, central hospital that presents extended travel distances for patients.

The intervention theories added valuable insights into stakeholders’ perceptions of challenges in routine outpatient treatment, their ideals for an enhanced clinical practice, and their assumptions about the impact of the digital health solution. Although the comparative analysis of intervention theories did not indicate irreconcilable conflicts across stakeholder groups at the 3 sites, it did reveal differences in stakeholder groups’ assumptions about the concept. The patients were generally very enthusiastic and envisioned greater flexibility with fewer consultations at the clinic, thus avoiding time taken to travel and to book and undergo regular blood tests. The health care professionals saw not only great advantages for patients but also challenges in relation to resources needed for implementing new practices in an already busy schedule, uncertainty as to which patient the concept would be the most relevant to, and how home monitoring would impact the patient population seen at the clinic. While physicians anticipated advantages from the extra clinical data collected, nurses anticipated additional tasks in assessing blood tests and PRO data. This was especially the case in UK, where PRO data are not routine and blood tests are usually assessed by the general practitioner. The nurses in CZ hoped for greater responsibility in assessing patient data, which is presently undertaken by physicians. Clinic managers could see financial, legal, and organizational challenges; however, they could also see the possibility of fewer face-to-face consultations, easing the limited physical capacity at the outpatient clinics.

The principle of reason of the concept was discussed in the consortium, enabling a common understanding of how the concept might be developed and implemented across the 3 countries. The results from the intervention theories also served as input to the design of the future outcome evaluation.

This contextual understanding informed the participatory design processes as well as the initial considerations for the organization and implementation of the solution. Finally, we found that field visits to the 3 clinical sites were of great value to the collaborative processes in the project. The value of interactive development of the design was acknowledged individually by all 3 countries, where both participating patients and health care professionals acquired inspiration from the presentation of considerations and solutions obtained at the other sites.

### Participatory Design Processes

During the workshops, patients and health care professionals gave feedback on the user interface and PoC devices. Patients had several ideas for improving the platform’s procedural flow, the joint assessment tool, and the PRO questionnaires. They also commented on the terminology used and stressed the importance of applying commonly used terms rather than technical terms and abbreviations. This feedback was essential to the further refinement of the user interface. The group and plenary discussions that followed these think aloud sessions gave us insights into patients’ and health care professionals’ immediate views on the platform and devices and on the digital health solution as a whole. The inclusion of both patients and health care professionals in the workshops was extremely useful, as this revealed the different perspectives of the 2 stakeholder groups and enriched the workshop discussions.

The laboratory facility allowed a short development turnover time for specific parts of the technologies involved. Patients were presented with visual mock-ups and working prototypes, and they provided valuable input in cooperation with a product designer. The information provided was used to create new prototypes and to fine-tune working scenarios. After testing several lancets available on the market for drawing blood, one was deemed appropriate for persons with reduced dexterity.

Initially, 2 PoC devices for analyzing blood samples were chosen for inclusion in the project. As neither device was at the time approved for patient self-testing at home, it was decided to test the cartridges used for blood sampling. The cartridge of one of the initially tested PoC devices was still under development and was changed on the basis of the feedback from patients. The cartridge of the other PoC device was found to be inappropriate for self-testing, and the device was thus replaced by 2 PoC devices, which further increased the complexity of the home-testing. As a result, we compared home-testing versus kiosk-testing (patients taking blood samples in a local setting, eg, general practitioners’ office or pharmacy, with health care professionals at hand), and the subsequent interviews with the patients led us to further considerations about how to organize the eHealth blood testing and indicated the need for more information and instructions. The patients and health care professionals testing the various devices gave valuable and necessary feedback to the developers of the equipment, which in the end saved the companies from futile investments in nonoperational processes.

### Feasibility Studies

The site user tests provided us with valuable understanding about the time needed for patient instruction prior to home monitoring and the handling of equipment and supplementary materials to take home. The tests also gave us insight into the types of challenges patients met when connecting to the ICT platform from home and performing the tasks allocated for the week.

Recording the extent and content of hospital help requested by patients helped us in the subsequent establishment of patient call centers. Log data provided information about patient compliance and how they completed the tasks allocated for the week. The majority of patients completed the tasks. Some patients had difficulties connecting to the ICT platform the first time, and others got confused by the short message service (SMS) text messages reminding each of the several tasks to be fulfilled on the same day. Despite commenting on the amount of time needed for examining, answering questions, and doing blood tests, patients were still in favor of testing at home and anticipated a possibility of recording RA-related problems in real time as an alternative to recounting them at the time of fixed visits to the clinic.

The comments made by patients in the notebook and illustrated by photos were extensive and very useful. Patients described their thoughts and experiences, for example, making room for the equipment at a desk and in the fridge, finding enough electric sockets, needing the help of a son and alike. One patient drew illustrations showing ideas for easy packaging and storage.

A report was produced for each site that described the lessons learned from the hospital and home environments. These reports were shared within the consortium and resulted in adjustments being made to resolve the reported issues before pilot testing.

Due to a need for testing changes and developments since the site user tests and to ensure time for further developments ahead of a clinical test scheduled for 2017, a pilot test was initiated in December 2016. Acknowledging the technical changes taking place right up to the start of the pilot study and the limited number of PoC devices available, it was decided to test in a series of 5 patients at a time. However, after the first 5 patients had completed the 3-week test, it was decided to discontinue the pilot study due to several technical challenges: (1) unstable internet connection, (2) missing SMS text messages to patients prior to tasks to be performed, (3) missing status updates and warnings to health care professionals when patients did not fulfill tasks, (4) failed connectivity between the PoC device and the ICT platform, (5) mechanical breakdown of one PoC device, and (6) final information material being too complex due to complicated PoC device instruction manuals.

These challenges resulted in patients not being able to connect to the ICT platform at various time points and video connections being unstable and of poor quality. The result was a disproportionate amount of resources used by patients calling the hotlines and the health care professionals trying to fix the technical challenges. The missing SMS text messages and status updates meant that some activities were not completed, and the comprehensive information material only seemed to add to the complexity of activities that patients were asked to perform. Despite this, at the subsequent interviews, the patients were still in favor of the digital health solution but pointed to a need for further development, the message being to make it simple and self-evident.

The feasibility study provided decisive insights into real-life use of the technology, including fundamental and necessary information about challenges and pitfalls in relation to the ICT platform and PoC devices and the information and instructions needed and how best to organize the workflow of the technology. Following an interim report after the first 5 patients had completed the pilot testing, a time-out was decided and a series of meetings and workshops were initiated within the consortium aimed at supporting and consolidating further development prior to the planned outcome evaluation.

## Discussion

### Principal Findings

The aim of the ELECTOR project was to develop and test a digital health solution for home-based monitoring of RA, including an ICT platform and supplementary components, across 3 different health care systems. Ongoing and systematic evaluation is important in the development of digital health solutions [16]. Evaluations of complex interventions need to be comprehensive and should include theoretical understanding and development work to ensure mutual learning [14,17]. Descriptions of the development process and the impact of ongoing evaluation also help to interpret the results of the intervention at a later stage [18]. The development process is ideally described as a chain of reasoning that leads from a statement of a problem to the definition of a solution and includes the following 7 steps of identification: drivers, visions, goal, objectives, requirements, design, and solution [16].

The vision of the ELECTOR project was to develop a more individualized treatment schedule that would reduce resource-demanding visits to the hospital for patients with RA with low disease activity, and this vision was developed and described during the funding application process [19]. The solution was conceptualized as a digital health solution including different components that were either under development or already developed but needed adjustment before use by patients with RA at home. Although the evaluation was in accordance with the steps recommended [16], a full requirement development process was not possible because the development of the solution and its components was already well advanced. Moreover, because the overall framework for creating this digital health solution was based on combining products and solutions from various companies and embedding them in a range of very different clinical settings, the formative evaluation thus served as an ongoing input that enabled learning as well as adaptation of the technical, clinical, visual, and organizational aspects of the concept.

The development of a technology that can accommodate daily practices of disease management across different settings requires an understanding of the complexity of the health care systems, organizational settings, and stakeholder groups involved as well as of the interplay between these and the technology in question [20,21]. The inclusion of 3 hospital sites representing different health care systems made it clear that the visualized solution can present different challenges in different health care systems, for example, in terms of responsibilities and competences of patients and health care professionals and the daily routines and collaborations. The use of field studies and intervention theories helped us to identify local visions and requirements related to the general implementation of the solution as well as those that were specific to the individual sites.

Numerous studies point to a multiplicity of barriers related to the implementation of digital initiatives among stakeholder groups [22,23]. The involvement of key stakeholders and real-life experiences is thus crucial. With the aim of developing a solution that would be relevant and would give added value to everyday practice, we established a consortium of stakeholders to ensure ongoing feedback to guide the development process. This input and feedback contributed to a wide range of adjustments in technical, visual, and practical components as well as adaptations in the conceptual design and organization of the digital health solution.

The learnings captured through these activities supported the development of the digital health solution and aided in identifying a “tipping point” [24] during a pilot study. Patients, health care professionals, and managers confirmed the relevance and value of the overall concept as well as the organizational setup. However, due to a range of challenges related to the technical components of the ICT platform, the pilot study came to a hold, allowing further development of the ICT platform and its components. Hence, the ongoing involvement of stakeholders and feedback to the consortium throughout the development process safeguarded us from initiating a large multicenter test at a time when the technology was in fact not ready.

### Strengths and Limitations

Although the evaluation was instrumental for the development of the digital health solution, it has some limitations. The project included several national clusters in differing clinical settings and related industries, and thus, the stakeholders were located in different countries and were not readily available. Due to the travel distances, the evaluation team was present only a few days at a time at the hospital clinics, and arranging interviews with busy patients and health care professionals was “the art of the possible.” As a result, interviews were performed both individually and in small groups. Furthermore, the laboratory facility was situated at a single location and involved a small number of patients from a specific treatment context.

The learning implied multinational and multicultural feedback, which was essential for the development of a fully operational set of tools for international use, including all aspects of a system for home-based monitoring of arthritis. Though limited by the inclusion of a relatively few patients and health care professionals, the site user tests were valuable by the diversity of the participating centers and gave insight into the technology’s implementation in routine clinical practice in several relevant hospital departments over the 3 countries representing much of the European health care traditions.

### Conclusions

This case study clearly demonstrated the advantages of conducting a formative evaluation early in the process to guide the development of a digital health solution. The formative evaluation was designed to inform the development process of a solution that aimed to be applicable and add value to everyday practice for patients and health care professionals in the treatment of RA in different European health care systems. Through a series of formative evaluation initiatives, an iterative process was implemented that included elucidating the inherent intervention theories of the digital health solution and of the patients, health care professionals, and managers and involving users in the design, testing, and adjustment of the solutions of the ICT platform and related components. The ongoing involvement of a range of relevant stakeholders helped to inform and influence the development of the solution. This process provided a basis for sequential formative testing and outcome evaluation and revealed its value in terms of providing constructive input and feedback, enabling the consortium to control the development process of a novel digital health solution.

## Appendix

Multimedia Appendix 1 An example of a matrix comparing the embedded intervention theory (“Principle of Reason”) of the eHealth project with intervention theories of patients with rheumatoid arthritis (RA) and healthcare professionals at one of the three sites.

## Abbreviations

ALT: alanine aminotransferase
Hgb: hemoglobin
hsCRP: high sensitivity C-reactive protein
ICTs: information and communication technologies
PoC: point-of-care
PROs: patient-reported outcomes
RA: rheumatoid arthritis
SMS: short message service
WBC: white blood cell

## References

[1] (2010). World Health Organization.

[2] Dinesen B, Nonnecke B, Lindeman D, Toft E, Kidholm K, Jethwani K, Young HM, Spindler H, Oestergaard CU, Southard JA, Gutierrez M, Anderson N, Albert NM, Han JJ, Nesbitt T (2016). Personalized Telehealth in the Future: A Global Research Agenda. J Med Internet Res.

[3] (2014). The Advisory Board.

[4] (2017). KORA.

[5] Mathijssen E, Vriezekolk J, Eijsbouts A, van DHF, van DBB (2018). Support needs for medication use and the suitability of eHealth technologies to address these needs: a focus group study of older patients with rheumatoid arthritis. Patient Prefer Adherence.

[6] Vos T, Flaxman AD, Naghavi M, Lozano R, Michaud C, Ezzati M, Shibuya K, Salomon JA, Abdalla S, Aboyans V, Abraham J, Ackerman I, Aggarwal R, Ahn SY, Ali MK, Alvarado M, Anderson HR, Anderson LM, Andrews KG, Atkinson C, Baddour LM, Bahalim AN, Barker-Collo S, Barrero LH, Bartels DH, Basáñez Maria-Gloria, Baxter A, Bell ML, Benjamin EJ, Bennett D, Bernabé Eduardo, Bhalla K, Bhandari B, Bikbov B, Bin AA, Birbeck G, Black JA, Blencowe H, Blore JD, Blyth F, Bolliger I, Bonaventure A, Boufous S, Bourne R, Boussinesq M, Braithwaite T, Brayne C, Bridgett L, Brooker S, Brooks P, Brugha TS, Bryan-Hancock C, Bucello C, Buchbinder R, Buckle G, Budke CM, Burch M, Burney P, Burstein R, Calabria B, Campbell B, Canter CE, Carabin H, Carapetis J, Carmona L, Cella C, Charlson F, Chen H, Cheng AT, Chou D, Chugh SS, Coffeng LE, Colan SD, Colquhoun S, Colson KE, Condon J, Connor MD, Cooper LT, Corriere M, Cortinovis M, de VKC, Couser W, Cowie BC, Criqui MH, Cross M, Dabhadkar KC, Dahiya M, Dahodwala N, Damsere-Derry J, Danaei G, Davis A, De LD, Degenhardt L, Dellavalle R, Delossantos A, Denenberg J, Derrett S, Des JDC, Dharmaratne SD, Dherani M, Diaz-Torne C, Dolk H, Dorsey ER, Driscoll T, Duber H, Ebel B, Edmond K, Elbaz A, Ali SE, Erskine H, Erwin PJ, Espindola P, Ewoigbokhan SE, Farzadfar F, Feigin V, Felson DT, Ferrari A, Ferri CP, Fèvre Eric M, Finucane MM, Flaxman S, Flood L, Foreman K, Forouzanfar MH, Fowkes FGR, Franklin R, Fransen M, Freeman MK, Gabbe BJ, Gabriel SE, Gakidou E, Ganatra HA, Garcia B, Gaspari F, Gillum RF, Gmel G, Gosselin R, Grainger R, Groeger J, Guillemin F, Gunnell D, Gupta R, Haagsma J, Hagan H, Halasa YA, Hall W, Haring D, Haro JM, Harrison JE, Havmoeller R, Hay RJ, Higashi H, Hill C, Hoen B, Hoffman H, Hotez PJ, Hoy D, Huang JJ, Ibeanusi SE, Jacobsen KH, James SL, Jarvis D, Jasrasaria R, Jayaraman S, Johns N, Jonas JB, Karthikeyan G, Kassebaum N, Kawakami N, Keren A, Khoo J, King CH, Knowlton LM, Kobusingye O, Koranteng A, Krishnamurthi R, Lalloo R, Laslett LL, Lathlean T, Leasher JL, Lee YY, Leigh J, Lim SS, Limb E, Lin JK, Lipnick M, Lipshultz SE, Liu W, Loane M, Ohno SL, Lyons R, Ma J, Mabweijano J, MacIntyre MF, Malekzadeh R, Mallinger L, Manivannan S, Marcenes W, March L, Margolis DJ, Marks GB, Marks R, Matsumori A, Matzopoulos R, Mayosi BM, McAnulty JH, McDermott MM, McGill N, McGrath J, Medina-Mora ME, Meltzer M, Mensah GA, Merriman TR, Meyer A, Miglioli V, Miller M, Miller TR, Mitchell PB, Mocumbi AO, Moffitt TE, Mokdad AA, Monasta L, Montico M, Moradi-Lakeh M, Moran A, Morawska L, Mori R, Murdoch ME, Mwaniki MK, Naidoo K, Nair MN, Naldi L, Narayan KMV, Nelson PK, Nelson RG, Nevitt MC, Newton CR, Nolte S, Norman P, Norman R, O'Donnell M, O'Hanlon S, Olives C, Omer SB, Ortblad K, Osborne R, Ozgediz D, Page A, Pahari B, Pandian JD, Rivero AP, Patten SB, Pearce N, Padilla RP, Perez-Ruiz F, Perico N, Pesudovs K, Phillips D, Phillips MR, Pierce K, Pion S, Polanczyk GV, Polinder S, Pope CA, Popova S, Porrini E, Pourmalek F, Prince M, Pullan RL, Ramaiah KD, Ranganathan D, Razavi H, Regan M, Rehm JT, Rein DB, Remuzzi G, Richardson K, Rivara FP, Roberts T, Robinson C, De LFR, Ronfani L, Room R, Rosenfeld LC, Rushton L, Sacco RL, Saha S, Sampson U, Sanchez-Riera L, Sanman E, Schwebel DC, Scott JG, Segui-Gomez M, Shahraz S, Shepard DS, Shin H, Shivakoti R, Singh D, Singh GM, Singh JA, Singleton J, Sleet DA, Sliwa K, Smith E, Smith JL, Stapelberg NJC, Steer A, Steiner T, Stolk WA, Stovner LJ, Sudfeld C, Syed S, Tamburlini G, Tavakkoli M, Taylor HR, Taylor JA, Taylor WJ, Thomas B, Thomson WM, Thurston GD, Tleyjeh IM, Tonelli M, Towbin JA, Truelsen T, Tsilimbaris MK, Ubeda C, Undurraga EA, van DWMJ, van OJ, Vavilala MS, Venketasubramanian N, Wang M, Wang W, Watt K, Weatherall DJ, Weinstock MA, Weintraub R, Weisskopf MG, Weissman MM, White RA, Whiteford H, Wiersma ST, Wilkinson JD, Williams HC, Williams SRM, Witt E, Wolfe F, Woolf AD, Wulf S, Yeh P, Zaidi AKM, Zheng Z, Zonies D, Lopez AD, Murray CJL, AlMazroa MA, Memish ZA (2012). Years lived with disability (YLDs) for 1160 sequelae of 289 diseases and injuries 1990-2010: a systematic analysis for the Global Burden of Disease Study 2010. Lancet.

[7] ELECTOR.

[8] Spradley J (1979). The ethnograpich interview. The ethnograpich interview.

[9] Hansen MB, Vedung E (2010). Theory-Based Stakeholder Evaluation. American Journal of Evaluation.

[10] Clemensen J, Larsen SB, Kyng M, Kirkevold M (2007). Participatory design in health sciences: Using cooperative experimental methods in developing health services and computer technology. Qual Health Res.

[11] Simonsen J, Robertson T( (2013). Routledge International Handbook of Participatory Design. Routledge International Handbook of Participatory Design.

[12] Simonsen J, Svabo C, Strandvad S, Samsom C, Hertzum M, Hansen O (2014). Situated Design Methods. Situated Design Methods.

[13] Jaspers MWM, Steen T, van DBC, Geenen M (2004). The think aloud method: a guide to user interface design. Int J Med Inform.

[14] Peters DH, Adam T, Alonge O, Agyepong IA, Tran N (2013). Implementation research: what it is and how to do it. BMJ.

[15] Hassling L, Nordfeldt S, Eriksson H, Timpka T (2005). Use of cultural probes for representation of chronic disease experience: exploration of an innovative method for design of supportive technologies. Technol Health Care.

[16] Catwell L, Sheikh Aziz (2009). Evaluating eHealth interventions: the need for continuous systemic evaluation. PLoS Med.

[17] Craig P, Dieppe P, Macintyre S, Michie S, Nazareth I, Petticrew M, Medical RCG (2008). Developing and evaluating complex interventions: the new Medical Research Council guidance. BMJ.

[18] Eysenbach G, CONSORT- E (2011). CONSORT-EHEALTH: improving and standardizing evaluation reports of Web-based and mobile health interventions. J Med Internet Res.

[19] Smith HR (2018). MedScape.

[20] Van VL, Wentzel J, Van GJE (2013). Designing eHealth that Matters via a Multidisciplinary Requirements Development Approach. JMIR Res Protoc.

[21] Castensøe-Seidenfaden Pernille, Reventlov HG, Teilmann G, Hommel E, Olsen BS, Kensing F (2017). Designing a Self-Management App for Young People With Type 1 Diabetes: Methodological Challenges, Experiences, and Recommendations. JMIR Mhealth Uhealth.

[22] Scott KC, Karem P, Shifflett K, Vegi L, Ravi K, Brooks M (2018). Evaluating barriers to adopting telemedicine worldwide: A systematic review. J Telemed Telecare.

[23] (2012). World Health Organization.

[24] Patton M (2011). Developmental evaluation: applying complexity concepts to enhance innovation and use. Developmental evaluation: applying complexity concepts to enhance innovation and use.

