# Supplementary figures and images for: The Development of Complex Digital Health Solutions: Formative Evaluation Combining Different Methodologies
Source: JMIR Res Protoc. 2018 Jul 16;7(7):e165. doi: 10.2196/resprot.9521 (PMC6066635; doi:10.2196/resprot.9521)

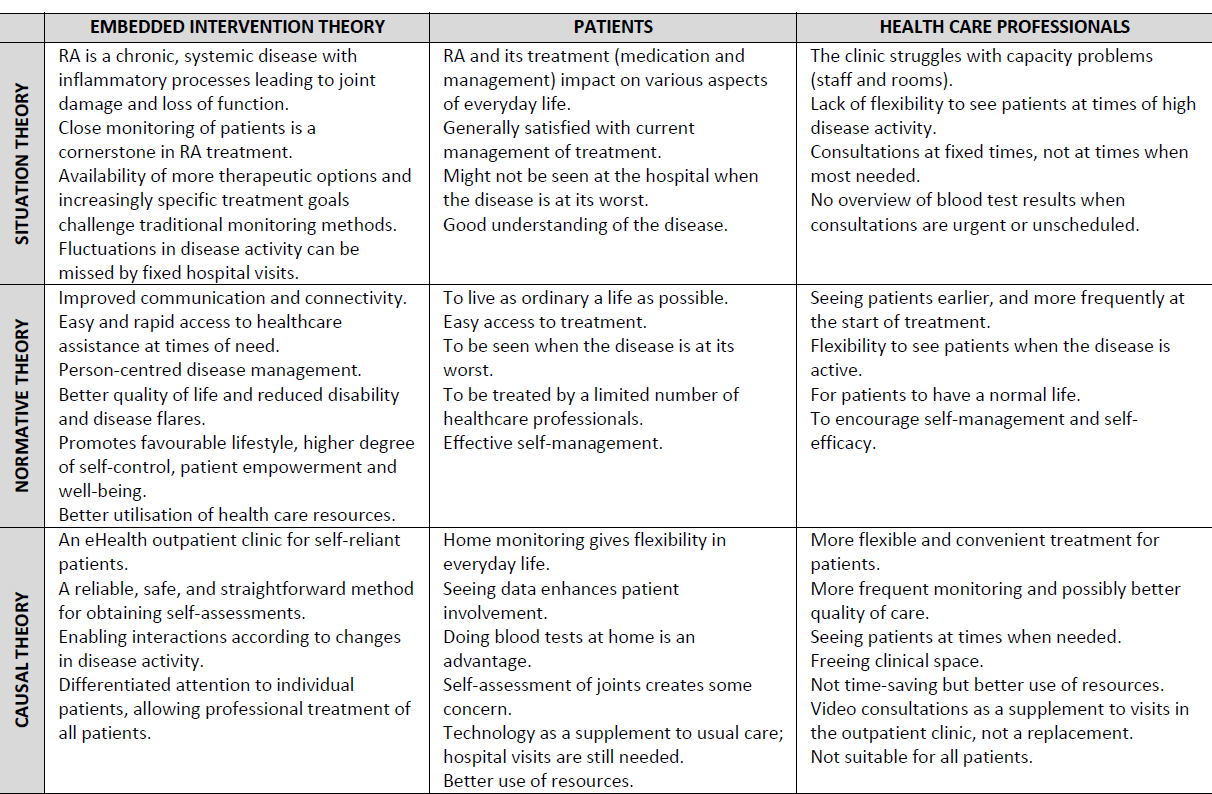

Supplement: Multimedia Appendix 1 [file resprot_v7i7e165_app1.PNG]
